# Slow gait speed – an indicator of lower cerebral vasoreactivity in type 2 diabetes mellitus

**DOI:** 10.3389/fnagi.2014.00135

**Published:** 2014-06-26

**Authors:** Azizah J. Jor’dan, Brad Manor, Vera Novak

**Affiliations:** ^1^Syncope and Falls in the Elderly Laboratory, Division of Gerontology, Department of Medicine, Beth Israel Deaconess Medical Center, Harvard Medical SchoolBoston, MA, USA; ^2^Institute for Aging Research, Hebrew SeniorLife, Harvard Medical SchoolBoston, MA, USA; ^3^Department of Neurology, Beth Israel Deaconess Medical Center, Harvard Medical SchoolBoston, MA, USA

**Keywords:** diabetes, gait, vasoreactivity, vasomotor, metabolic

## Abstract

**Objective:** Gait speed is an important predictor of health that is negatively affected by aging and type 2 diabetes. Diabetes has been linked to reduced vasoreactivity, i.e., the capacity to regulate cerebral blood flow in response to CO_2_ challenges. This study aimed to determine the relationship between cerebral vasoreactivity and gait speed in older adults with and without diabetes.

**Research design and methods:** We studied 61 adults with diabetes (65 ± 8 years) and 67 without diabetes (67 ± 9 years) but with similar distribution of cardiovascular risk factors. Preferred gait speed was calculated from a 75 m walk. Global and regional perfusion, vasoreactivity and vasodilation reserve were measured using 3-D continuous arterial spin labeling MRI at 3 Tesla during normo-, hyper- and hypocapnia and normalized for end-tidal CO_2_.

**Results:** Diabetic participants had slower gait speed as compared to non-diabetic participants (1.05 ± 0.15 m/s vs. 1.14 ± 0.14 m/s, *p* < 0.001). Lower global vasoreactivity (*r*^2^_adj_ = 0.13, *p* = 0.007), or lower global vasodilation reserve (*r*^2^_adj_ = 0.33, *p* < 0.001), was associated with slower walking in the diabetic group independently of age, BMI and hematocrit concentration. For every 1 mL/100 g/min/mmHg less vasodilation reserve, for example, gait speed was 0.05 m/s slower. Similar relationships between vasodilation reserve and gait speed were also observed regionally within the cerebellum, frontal, temporal, parietal, and occipital lobes (*r*^2^_adj_ = 0.27–0.33, *p* < 0.0001). In contrast, vasoreactivity outcomes were not associated with walking speed in non-diabetic participants, despite similar vasoreactivity ranges across groups.

**Conclusion:** In the diabetic group only, lower global vasoreactivity was associated with slower walking speed. Slower walking in older diabetic adults may thus hallmark reduced vasomotor reserve and thus the inability to increase perfusion in response to greater metabolic demands during walking.

## INTRODUCTION

Gait speed is predictive of mobility, morbidity, and mortality in older adults (Guralnik et al., 1995; Studenski et al., 2011). Vasoreactivity is an important cerebrovascular control mechanism used to maintain brain perfusion during increased metabolic demands (Bullock et al., 1985; Schroeder, 1988) such as walking, and can be clinically quantified by the vasodilation responses to hypercapnia (Low et al., 1999; Lavi et al., 2006). In healthy older adults, blood flow velocities in the middle cerebral artery territory, which supplies numerous brain regions involved in locomotor control, increased proportionally to walking speed (Novak et al., 2007). In a population-based study comprising community-dwelling older adults both with and without risk factors for falls (e.g., diabetes, stroke, use of walking aids, etc.), slower walkers exhibited lower vasoreactivity within the middle cerebral artery territory as measured by Transcranial Doppler ultrasound (Sorond et al., 2010). Slowing of gait may thus reflect an early manifestation of underlying abnormalities in vasoreactivity and perfusion adaptation to the metabolic demands of walking. However, the relationship between brain vascular health and walking has not yet been established.

Type 2 diabetes accelerates brain aging (Biessels et al., 2002; Last et al., 2007) and has also been linked with microvascular disease and altered cerebral blood flow regulation (Allet et al., 2008; Várkuti et al., 2011) and vasoreactivity (Novak et al., 2011). Diabetes is associated with reduced gait speed and related functional decline (Volpato et al., 2010). In older adults, gait characteristics have been linked to gray matter atrophy and white matter hyperintensities (Rosano et al., 2007a,b; Callisaya et al., 2013). Moreover, gray matter atrophy appears to have a stronger effect on locomotor control in those with type 2 diabetes as compared those without, suggesting that the control of walking may be more dependent upon supraspinal control within this population (Manor et al., 2012). This study therefore aimed to determine the relationship between vasoreactivity and gait speed in older adults with and without type 2 diabetes. We hypothesized that lower global and regional vasoreactivity would be associated with slower gait speed in older adults, particularly in those with type 2 diabetes.

## MATERIALS AND METHODS

### PARTICIPANTS

This secondary analysis was completed on prospectively collected data from community-dwelling older adults originally recruited via local advertisement. We analyzed records from three completed projects spanning March 2003–July 2012: Cerebral vasoregulation in the elderly with stroke (March 2003–April 2005); Cerebral perfusion and cognitive decline in type 2 diabetes (January 2006–December 2009); and Cerebromicrovascular disease in elderly with diabetes (August 2009–July 2012). Grant numbers are provided in the study funding section.

Collectively, these three studies recruited 447 participants who signed informed consent (212 non-diabetics, 151 diabetics, 84 stroke). 213 participants (103 non-diabetics, 69 diabetics, 41 stroke) were excluded at that time for the following reasons: (1) ineligible after the screening visit (*n* = 117); (2) withdrew consent (*n* = 31); (3) lost to follow-up (*n* = 13); (4) study terminated (*n* = 52) for reasons related to exclusion criteria or other reasons such as lack of permission from primary care provider, no transcranial Doppler insonation window, unstable/untreated hypertension, high BMI, cerebral palsy, claustrophobia, atrial fibrillation, inappropriate behavior during screening, metal implant, abdominal pain due to kidney stone, or entered a nursing home.

For the present analysis, we excluded an additional 43 stroke records that met the exclusion criteria for the current analyses, 34 records that did not have complete datasets, and 29 records from subjects who completed more than one of the above-mentioned studies. In each of the latter cases, the most recent record was kept. Thus, records from a total of 128 subjects were included in the present analysis.

Participants were originally screened by medical history and physical, neurological, and laboratory examinations. Research protocols were conducted in accordance with the ethical standards of the Beth Israel Deaconess Medical Center (BIDMC) Clinical Research Center and all participants signed an informed consent, as approved by the Institutional Review board at BIDMC.

The diabetic group included men and women aged 50–85 years with a physician diagnosis and treatment of type 2 diabetes mellitus with oral agents and/or combinations with insulin for at least one year. Diabetes treatments included insulin, oral glucose-control agents (sulfonylurea, second generation agents), their combinations and diet. Non-diabetic participants had no history of metabolic disorder and were recruited to match the age and gender characteristics of the diabetic group (**Table 1**).

**Table 1:** Demographic characteristics of the non-diabetic and diabetic groups.

	Non-diabetic group	Diabetic group	*p*
N	67	61	
Age (years)	67 ± 9	65 ± 8	NS
Sex (women, %)	59	49	NS
Body Mass Index (kg/m^2^)	25.6 ± 4	29.1 ± 5	< 0.0001
Mini-Mental State Exam (1–30)	28.2 ± 1.8	28.2 ± 1.8	NS
Diabetes duration (years)	–	12.7 ± 9	–
Systolic blood pressure (mmHg)	130.6 ± 10.8	133.5 ± 8.5	NS
Diastolic blood pressure (mmHg)	68.5 ± 8.4	71 ± 7.6	NS
Hypertension (yes/no)	20/47	38/23	0.0003
Peripheral neuropathy (%)	18	51	0.001
Hyperlipidemia (yes/no)	7/60	34/27	< 0.0001
Gait speed (m/s)	1.14 ± 0.14	1.05 ± 0.15	0.0004
Rating of perceived exertion (1–10)	1.49 ± 1.43	2.17 ± 2.13	0.0386
Global gray matter (cm^3^)	639 ± 82	620 ± 62	NS
Global white matter (cm^3^)	436 ± 56	424 ± 52	NS
Global white matter hyperintensities (cm^3^)	11 ± 7	13 ± 7	NS
Global vasoreactivity (mL/100g/min/mmHg)	0.98 ± 0.09	1.10 ± 0.09	NS
Global vasodilation reserve (mL/100g/min/mmHg)	0.35 ± 1.7	0.44 ± 1.7	NS
Global vasoconstriction reserve (mL/100g/min/mmHg)	1.5 ± 3.2	1.4 ± 2.5	NS
Hemoglobin A1c (%)	5.7 ± 0.3	7.3 ± 1.3	< 0.0001
Hematocrit (%)	40.4 ± 3.7	39.3 ± 3.7	NS
Fasting glucose (mg/dL)	84.7 ± 12.3	121.7 ± 43.1	< 0.0001
Total cholesterol (mg/dl)	194 ± 36	166 ± 38.8	< 0.0001
Cholesterol-to-HDL ratio	3.4 ± 0.9	3.4 ± 1.2	NS
Triglycerides (mg/dl)	130.2 ± 70	146 ± 94.6	NS

*Data = means ± SD unless otherwise indicated. *p* = between-group comparisons. NS = non-significant.*

Exclusion criteria for the current analysis were history of stroke, myocardial infarction, clinically significant arrhythmia or other cardiac disease, nephropathy, severe hypertension (i.e., systolic BP > 200, diastolic BP > 110 mm Hg or the use of three or more antihypertensive medications), seizure disorder, kidney or liver transplant, renal disease, any other neurological or systemic disorder (aside from peripheral neuropathy), and current recreational drug or alcohol abuse. MRI exclusion criteria were incompatible metal implants, pacemakers, arterial stents, claustrophobia and morbid obesity (i.e., BMI > 40).

### PROTOCOL

Participants completed medical history, autonomic symptoms, and physical activity questionnaires. A study physician completed physical, neurological, and ophthalmologic examinations. None of the study participants had active foot ulcers during the study. A study nurse completed a fasting blood draw and recorded vital signs, anthropometric and adiposity measures. Participants also completed a comprehensive cognitive exam, autonomic testing, perfusion MRI of the brain and a gait assessment. For this study, we focused analyses on gait and MRI-based measures of cerebral perfusion and vasoreactivity.

#### Walking test

A 12-min walk was completed along a 75 m course on an 80 m × 4 m indoor hallway. Participants were instructed to walk at preferred speed (i.e., a pace they deemed as comfortable or normal), which has excellent test–retest reliability, even in those with severe diabetic complications (Steffen et al., 2002; Manor et al., 2008). The time taken to complete each 75 m length and total distance were recorded. For the present analysis, we only examined data from the first hallway length (i.e., the first 75 m of the trial) in order to minimize potential confounders of turning and fatigue. Assistive devices were not used for ambulation. A rating of perceived exertion was asked of the participant before the start of the walk and once the walk was completed. Rating of perceived exertion ranged from 0 (no exertion) to 10 (very, very strong exertion).

#### Magnetic resonance imaging (MRI)

Brain imaging was completed in a 3T GE HDx MRI scanner (GE Medical Systems, Milwaukee, WI, USA) within the Center for Advanced MR Imaging at the BIDMC. 3D spiral continuous arterial spin labeling (CASL) MRI was used to quantify cerebral perfusion (Alsop and Detre, 1998; Detre et al., 1998; Floyd et al., 2003) during normocapnia, hypocapnia, and hypercapnia. Vasoreactivity was assessed as perfusion responses to vasodilation during hypercapnia and vasoconstriction to hypocapnia (Kety and Schmidt, 1948), as a non-invasive reliable method of assessing the integrity of cerebral vasculature (Fujishima et al., 1971; Yen et al., 2002). Specifically, two-minute scans were acquired during normal breathing (i.e., baseline normocapnia; end tidal CO_2_ concentration 33–38 mmHg), hyperventilation (i.e., hypocapnia; participants hyperventilated to reduce CO_2_ to a target of 25 mmHg), and rebreathing (i.e., hypercapnia; participants breathed a mixture of 5% CO_2_ and 95% air to increase CO_2_ to a target of 45 mmHg).

Respiratory rate, tidal volume and end-tidal CO_2_ values were measured during each scan using an infrared end-tidal volume gas monitor (Capnomac Ultima, General Electric, Fairfield, CT, USA) attached to a face-mask. Blood pressure and heart rate were also recorded at one-minute intervals using an upper-arm automatic blood pressure cuff and finger photoplethysmogram.

Perfusion images were acquired using a custom 3D CASL sequence (*T*_R_/*T*_E_ = 10.476/2.46 ms, Label duration = 1.45 s, post-label delay = 1.525 s, with 64 × 64 matrix in the axial plane and 40 slices with thickness = 4.5 mm, seven spiral interleaves and the bandwidth = 125 kHz). Images were averaged over each condition to maximize signal-to-noise ratio.

A T1-weighted MP-RAGE structural imaging sequence was completed and used for registration of CASL images. Imaging parameters were: TE/TR = 3.3/8.1 ms, flip angle of 10°, 1–3 mm slice thickness, 24 cm × 19 cm field of view (FOV), 256 × 192 matrix size.

### DATA ANALYSIS

#### Gait speed

Average gait speed (m/s) was computed from the first 75 m of walking by dividing distance by time. This valid and reliable outcome predicts future health status and functional decline in numerous older adult populations (Quach et al., 2011; Studenski et al., 2011).

#### Image analysis

A rigid-body model (Collignon et al., 1995; Wells et al., 1996) was used for registration of the MP-RAGE image on CASL images using the Statistical Parametric Mapping software package (SPM, Wellcome Department of Imaging Neuroscience, University College, London, UK). This “normalization” module was employed to stereotactically normalize structural images to a standard space defined by ideal template image(s). The registered perfusion image was then overlaid on the segmented anatomical regions to obtain regional perfusion measurements. Generated maps of gray matter and white matter were segmented based upon the LONI Probabilistic Brain Atlas (Shattuck et al., 2008) and was used to calculate global volumes. All image segmentations were completed using Interactive Data Language (IDL, Research Systems, Boulder, CO, USA) and MATLAB (MathWorks, Natick, MA, USA) software.

#### Perfusion analyses

Perfusion and vasoreactivity were calculated in five regions-of-interest: the cerebellum, frontal, temporal, parietal, and occipital lobe. Within each region, perfusion was normalized for tissue volume and thus expressed in mL/100 g/min. Four perfusion measures were calculated for each region: baseline perfusion during normal breathing, cerebral vasoreactivity, vasodilation reserve, and vasoconstriction reserve. Each outcome was computed globally and within each brain region-of-interest.

Perfusion values were normalized to each subject’s average CO_2_ level during this condition. Vasoreactivity measures were calculated as previously described (Last et al., 2007; Hajjar et al., 2010; Novak et al., 2011). Briefly, vasoreactivity was defined as the slope of the best-fit line produced by linear regression of perfusion and CO_2_ values across the three conditions (i.e., normal breathing, CO_2_ rebreathing, and hyperventilation). Vasodilation reserve was defined as the increase in perfusion from baseline to the rebreathing condition, normalized to the change in CO_2_ between these two conditions. Vasoconstriction reserve was defined as the decrease in perfusion from baseline to the hyperventilation condition, normalized to the change in CO_2_ between these two conditions.

### STATISTICAL ANALYSIS

All analyses were performed using JMP software (SAS Institute, Cary, NC, USA). Descriptive statistics were used to summarize all variables. Outcomes have been expressed as either the mean ± SD or categorical (yes/no) for each group. Student’s *t*, Fisher’s Exact and Chi-squared tests were used to compare group demographics.

We examined the effects of diabetes on both perfusion measures and gait speed using ANCOVA. For perfusion measures, the model effect was group and covariates included age, hematocrit (Hct) concentration and hypertension. Hct was included because it is inversely correlated with blood viscosity and is higher in men than women (Wells and Merrill, 1962; Kameneva et al., 1999; Zeng et al., 2000). Hypertension was included as a covariate because it affects small blood vessels of the body and may therefore alter cerebral blood flow regulation (Alexander, 1995; Hajjar et al., 2010). For gait speed, the model effect was group and covariates included age, gender and BMI.

Linear least-square regression analyses were used to test the hypotheses that (1) those with lower vasoreactivity demonstrate slower preferred gait speed, and (2) this association between vasoreactivity and gait speed is stronger (as reflected in the correlation coefficient, *r*^2^_adj_) in older adults with diabetes as compared to those without diabetes. The dependent variable was gait speed. Model effects included perfusion outcome, group (non-diabetic, diabetic), and their interaction. Separate models were performed for each global and regional perfusion and vasoreactivity outcome. Age, BMI, and Hct concentration were included as covariates. Significance level was set to *p* = 0.05 for each global perfusion and vasoreactivity outcome. The Bonferroni-adjusted significance level for multiple comparisons (*p* = 0.01) was used to determine significance of models examining outcomes within each of the five brain regions-of-interest.

## RESULTS

### PARTICIPANTS

Groups were matched by age and gender and had a similar cardiovascular risk factors (e.g., blood pressure, triglycerides, cardiovascular disease history), yet the diabetic group had higher BMI (*p* < 0.0001). The prevalence of hypertension and peripheral neuropathy was also higher in the diabetic group as compared to the non-diabetic group (62% vs. 30%, *p* < 0.001 and 51% vs. 18%, *p* < 0.001, respectively). Participants with diabetes had greater HbA1c and serum glucose levels, but lower total cholesterol as compared to the non-diabetic group. Blood Hct concentration was similar between groups, but overall, higher in males as compared to females (42% vs. 38%, *p* < 0.001). Groups did not differ in global gray matter, white matter or white matter hyperintensity volumes (see **Table 1**).

### THE EFFECTS OF DIABETES ON PERFUSION AND CEREBRAL VASOREACTIVITY

#### Baseline perfusion and cerebral vasoreactivity

The diabetic and non-diabetic groups had similar global and regional perfusion at baseline after normalizing for baseline CO_2_ levels and adjusting for age, Hct concentration and the presence of hypertension. Global and regional vasoreactivity, as well as vasodilation and vasoconstriction reserve, were also similar between groups (**Table 1**).

### THE EFFECTS OF DIABETES ON GAIT SPEED

The diabetic group had slower preferred gait speed as compared to the non-diabetic group (1.05 ± 0.15 m/s vs. 1.14 ± 0.14 m/s, *p* < 0.001; **Table 1**). This group difference remained significant (*p* = 0.007) after adjusting for age, gender, and BMI.

Across all participants, those with higher BMI had slower gait speed (*r*^2^_adj_ = 0.04, *p* = 0.01). Specifically, within the diabetic group, those with higher fasting glucose had slower gait speed (*r*^2^_adj_ = 0.13, *p* = 0.003). Gait speed was not correlated with the participant’s rating of perceived exertion, HbA1c levels or diabetes diagnosis duration. The diabetic group had a higher change in rating of perceived exertion (i.e., difference from the start of walk from the end of the walk) compared to the non-diabetic group (2.17 ± 2.13 vs. 1.49 ± 1.43, *p* = 0.039).

### RELATIONSHIPS BETWEEN CEREBRAL VASOREACTIVITY AND GAIT SPEED

#### Cerebral vasoreactivity

**FIGURE 1:**
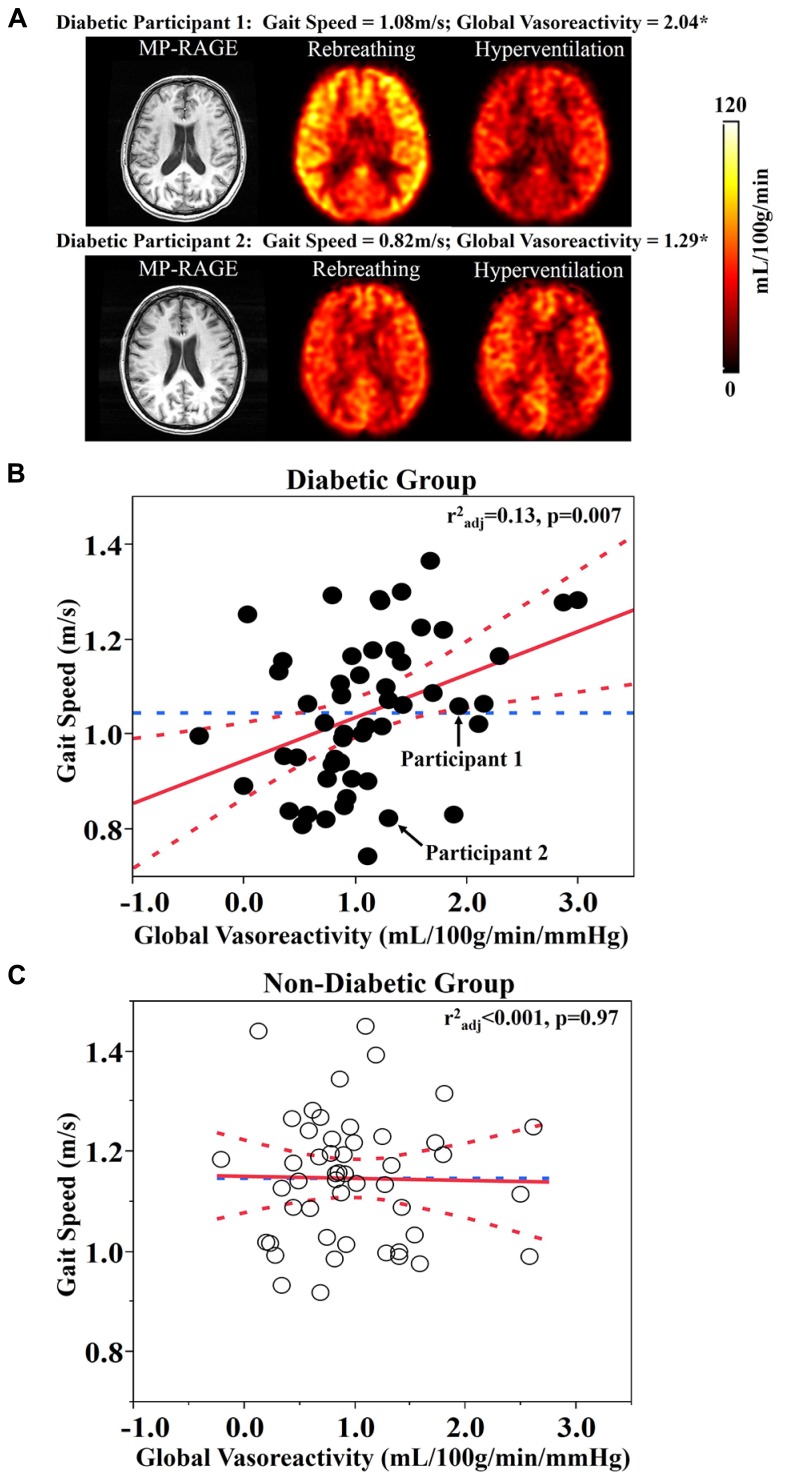
**(A)** Reconstructed anatomical (i.e., MP-RAGE) and perfusion maps for two participants with type 2 diabetes mellitus. The top row represents a participant with diabetes that has high global vasoreactivity and fast gait speed (see Diabetic Participant 1 in **A**). The bottom row represents a participant with diabetes that has low global vasoreactivity and slow gait speed (see Diabetic Participant 2 in **A**). **(B)** The relationship between global vasoreactivity and gait speed in the diabetic group. **(C)** The relationship between global vasoreactivity and gait speed in the non-diabetic group. Vasoreactivity was calculated as the change in perfusion from hypocapnia (hyperventilation) to hypercapnia (CO_2_ rebreathing) conditions, normalized to the change in CO_2_ values. Best fit – red solid line; Confidence Intervals – red dotted lines; Gait speed mean – blue dotted line; *unit – mL/100g/min/mmHg.

Least square models revealed that global vasoreactivity was related to gait speed, but that this relationship was dependent upon group (*F*_1,96_ = 5.48, *p* = 0.024). This group by vasoreactivity interaction was independent of age, BMI, and Hct levels. *Post hoc* testing indicated that within the diabetic group, those with lower global vasoreactivity walked more slowly (*r*^2^_adj_ = 0.13, *p* = 0.007; **Figures 1A,B**). In the non-diabetic group, however, global vasoreactivity was not correlated with gait speed (**Figure 1C**). A trend towards a similar interaction was also observed between frontal lobe vasoreactivity and group (*F*_1,95_ = 4.32, *p* = 0.04); that is, in the diabetic group *only*, those with lower frontal lobe vasoreactivity tended to walk slower (*r*^2^_adj_ = 0.13, *p* = 0.007). Yet, this interaction was not significant based upon the Bonferroni-adjusted significance level (*p* = 0.01).

#### Vasodilation reserve

**FIGURE 2:**
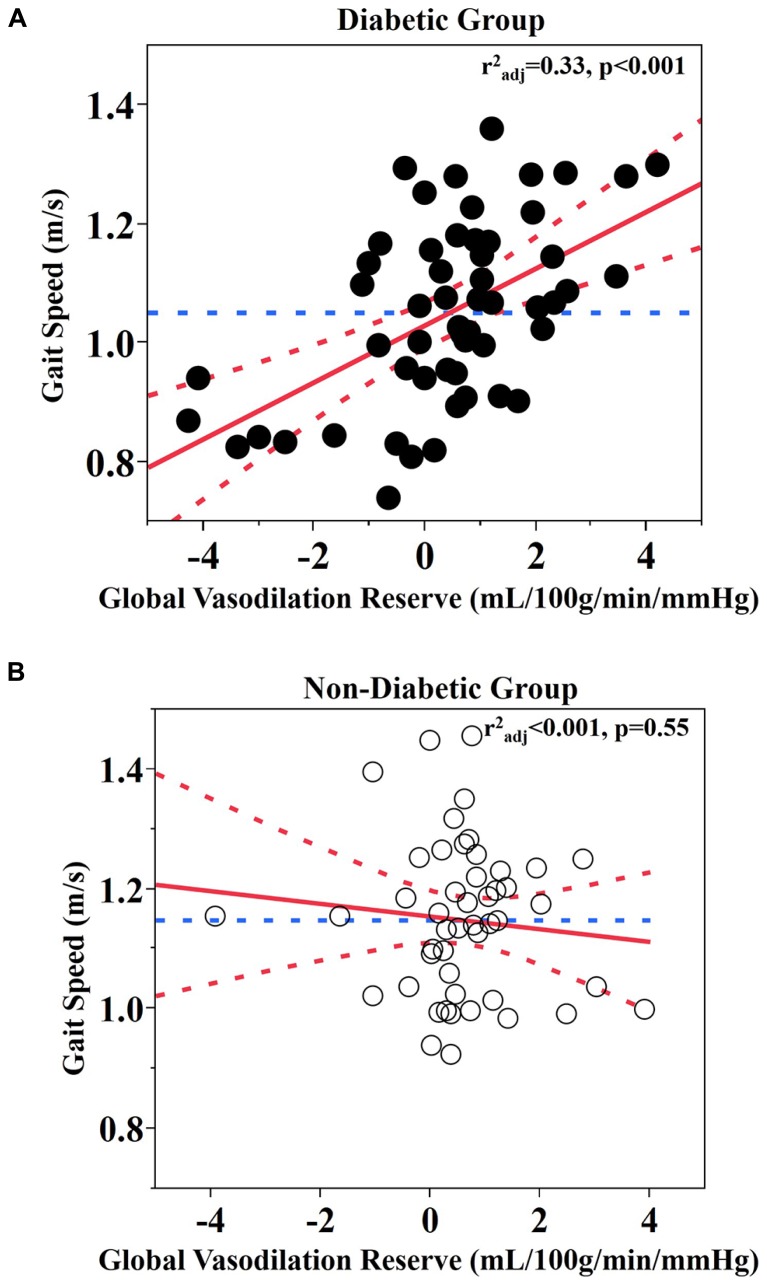
**Relationship between global vasodilation reserve and gait speed. (A)** Diabetic group **(B)** Non-diabetic group. Best fit – red solid line; Confidence bounds – red dotted lines; Gait speed mean – blue dotted line.

Least square models revealed a significant relationship between global vasodilation reserve and gait speed, but that this relationship was also dependent upon group (*F*_1,97_ = 12, *p* < 0.001). This significant interaction between group and vasodilation reserve was independent of age, BMI, and Hct levels. *Post-hoc* testing revealed that within the diabetic group *only*, those with lower global vasodilation reserve walked more slowly (*r*^2^_adj_ = 0.33, *p* < 0.0001; **Figure 2A**).

Similar interactions were present between group and vasodilation reserve within each brain region-of-interest (cerebellum: *F*_1,94_ = 13, *p* < 0.001; frontal lobe: *F*_1,96_ = 8.49, *p* = 0.005; temporal lobe: *F*_1,96_ = 17.1, *p* < 0.001; parietal lobe: *F*_1,95_ = 8.72, *p* = 0.004; occipital lobe: *F*_1,95_ = 8.99, *p* = 0.004). In each case, within the diabetic group *only*, those with lower vasodilation reserve walked slower (Least square: *r*^2^_adj_ = 0.27–0.33, *p* ≤ 0.001; **Table 2**). In the non-diabetic group, neither global nor regional vasodilation reserve was correlated with gait speed (**Figure 2B**).

**Table 2:** Vasodilation reserve and gait speed relationship in the diabetic group.

	*M*	*r*^2^_adj_	*p*
Gait speed (m/s) Vasodilation reserve (mL/100g/min/mmHg)	1.05 ± 0.02		
Global	0.42 ± 0.2	0.33	<0.0001
Cerebellum	0.62 ± 0.2	0.33	<0.0001
Frontal	0.31 ± 0.3	0.27	<0.0001
Temporal	0.48 ± 0.2	0.33	<0.0001
Parietal	0.32 ± 0.3	0.30	<0.0001
Occipital	0.42 ± 0.3	0.29	<0.0001

*Data are least square means (M) ± SE, *r*^2^_adj_ and *p* value adjusted for age, BMI, and Hct.*

#### Vasoconstriction reserve

Global and regional vasoconstriction was not related to gait speed in either group.

#### Baseline perfusion

Global or regional baseline perfusion was not related to gait speed within either group.

#### Additional covariates

Secondary analyses were performed to determine if within the diabetic group, the observed relationships between cerebral blood flow regulation outcomes and gait speed were influenced by the participant’s height, weight, rating of perceived exertion, the burden of white matter hyperintensities, or the prevalence of hypertension or peripheral neuropathy. In each case, relationships between cerebral blood flow regulation and gait speed remained significant after adjusting for potential covariance associated with these factors.

## DISCUSSION

This study has shown that within the diabetic group, those with lower global vasoreactivity walked more slowly. Our results further indicate that within this group, vasodilation reserve, or the capacity to increase cerebral perfusion specifically in response to hypercapnia, was linked to gait speed, which is an overall measure of health in older adults. This relationship was observed both globally and within each brain region-of-interest (i.e., cerebellum, frontal lobe, temporal lobe, parietal lobe, and occipital lobe). Specifically, for every 1 mL/100 g/min/mmHg less global vasodilation reserve, gait speed was 0.05 m/s slower in the diabetic group. These relationships were independent of age, BMI, Hct, and additional covariates (i.e., height, weight, rating of perceived exertion, white matter hyperintensities, and the prevalence of hypertension or peripheral neuropathy).

Both groups presented with average walking speeds that were slower than published norms; i.e., 1.2–1.4 m/s for healthy adults over 50 years of age (Bohannon, 1997). Diabetic participants walked 0.09 ± 0.15 m/s more slowly than those without diabetes, which reflects a clinically significant difference between groups (Kwon et al., 2009). In the diabetic group, walking speed was correlated with fasting glucose levels, but not with diabetes duration or HbA1c. Furthermore, as can be observed in **Figure 2A**, several participants with diabetes that walked the slowest appeared to have abnormal responses to the hypercapnia condition (i.e., no change or decreased perfusion). For these individuals, this response may function as a compensatory response to ensure adequate perfusion even during resting conditions (Novak et al., 2006).

Previous research in older adults has linked slow gait speed to impaired “neurovascular coupling,” or the change in cerebral blood flow in response to the performance of a cognitive task (Girouard and Iadecola, 2006; Iadecola and Nedergaard, 2007; Sorond et al., 2011). For example, Sorond et al. (2011) investigated the association between gait speed and neurovascular coupling as quantified by the change in blood flow velocity within the middle cerebral artery (using Transcranial Doppler Ultrasonography) in response to performance of the *n-*back cognitive task. Those with impaired neurovascular coupling walked more slowly. They also reported an interaction between neurovascular coupling and white matter hyperintensity burden, such that the presence of white matter hyperintensities was associated with reduced gait speed, *except* in those individuals with relatively strong neurovascular coupling. Previous work by Novak et al. (2007, 2011) further demonstrated that lower vasoreactivity is linked to reduced gait speed independently of white matter hyperintensities specifically within older adults with type 2 diabetes. Therefore, neurovascular coupling appears to one mechanism that links vascular changes to neuronal activity, and is therefore essential for the preservation of functional outcomes. This notion is in line with the “brain reserve” hypothesis (Bullock et al., 1985; Stern, 2002) and may help explain the results of the current study. In other words, while diabetes was associated with reduced gait speed overall, those diabetic participants with greater vasoreactivity (or vasodilation reserve) tended to walk at similar speeds as non-diabetic controls.

Walking is a complex act that requires the coordination of locomotor, cardiovascular, and autonomic systems. The lack of relationship between cerebral vasoreactivity and gait speed in those without diabetes is supported by the notion that gait is largely autonomous and governed primarily by supraspinal elements of the motor control system under normal or healthy conditions (Stoffregen et al., 2000; Manor et al., 2010; Kloter et al., 2011). In those with diabetes, however, the capacity to modulate cerebral perfusion between conditions of hyper- and hypocapnia (i.e., vasoreactivity, a widely used prognosis of metabolic cerebral blood flow regulation) was associated with gait speed. These results suggest that in diabetic patients, the regulation of walking speed is dependent upon cerebral elements related to the locomotor control system. This notion is supported by research demonstrating that walking requires adjustments of the cardiovascular and cerebrovascular systems that are coordinated to increase blood pressure and cerebral blood flow velocities in order to meet metabolic demands (Novak et al., 2007; Perrey, 2013). Therefore, those diabetic participants with reduced vasoreactivity may have a diminished ability to increase perfusion in response to the metabolic demand associated with walking.

The relationship between vasoreactivity and gait speed that was observed in the diabetic group, but not in the non-diabetic group might also be explained by the complex effects of diabetes on cerebral vasculature and metabolism. Diabetes accelerates aging in the brain (Launer, 2006) and alters vascular reactivity through the combined effects of central insulin resistance on microvasculature, brain metabolism, glucose utilization, and neuronal survival. Central insulin plays an important role as a neuromodulator in key processes such as cognition (Shemesh et al., 2012; Freiherr et al., 2013), energy homeostasis, and glucose utilization during activity (e.g., walking). Cerebral insulin may directly modulate neuron–astrocyte signaling through neurovascular coupling and autonomic control of vascular tone and thus enable better regulation of local and regional perfusion (Lok et al., 2007) and neuronal activity in response to various stimuli (Amir and Shechter, 1987; Cranston et al., 1998; Kim et al., 2006; Muniyappa et al., 2007) including walking. Type 2 diabetes decreases insulin sensitivity in the brain, insulin transport through the blood–brain barrier, and insulin receptor’s sensitivity, and it alters glucose metabolism and energy utilization (Plum et al., 2005, 2006; Hallschmid et al., 2007; Freiherr et al., 2013). Glucotoxicity and endothelial dysfunction associated with chronic hyperglycemia further affect perfusion, vasoreactivity, and metabolism (Makimattila and Yki-Jarvinen, 2002; Brownlee, 2005; Kilpatrick et al., 2010) and contribute to neuronal loss (Manschot et al., 2006, 2007; Last et al., 2007). Therefore, inadequate insulin delivery to brain tissue combined with altered energy metabolism may affect neuronal activity in multiple regions, but in particular the motor and cognitive networks that have high demands on energy (Gunning-Dixon and Raz, 2000). Diabetes may therefore especially alter neuronal activity and energy utilization during complex tasks like walking which require coordination of neuronal activity in numerous brain regions. As such, even if the same amount of blood flow is delivered to the neurons, energy utilization may be reduced in diabetic as compared to non-diabetic brain, leading to reduced neuronal activity and function, such as walking speed.

While our study controlled for numerous variables associated with gait speed, it did not control for other associated variables, such as muscular strength or fear of falling (Bendall et al., 1989; Chamberlin et al., 2005). The current study has the advantage of investigating regional perfusion in response to CO_2_ challenges using 3-D CASL MRI; however, the measures were recorded while participants were lying supine and not during walking. Although these regional perfusion measures may be lost, future studies are warranted to utilize wireless cerebral blood flow measurement tools (e.g., portable TCD or functional near-infrared spectroscopy) to examine the effects of diabetes on cerebral perfusion when walking at different speeds. Moreover, this is a cross-sectional study and thus, observed relationships between low vasoreactivity and slow gait speed does not necessarily imply a causal link between the two. As such, prospective studies are needed to determine potential mechanisms underlying the observed relationship between vasoreactivity and gait speed in those with diabetes, the predictive value of vasoreactivity as a clinical tool, and the potential for therapies targeting cerebral blood flow regulation to improve functional outcome in this vulnerable population.

## AUTHOR CONTRIBUTIONS

Azizah J. Jor’dan analyzed the data, performed statistical analyses and wrote the manuscript. Brad Manor oversaw statistical analyses, data interpretation and contributed to manuscript preparation. Vera Novak designed the study, conducted experiments, and oversaw all aspects of the study, data interpretation and manuscript preparation.

## Conflict of Interest Statement

The authors declare that the research was conducted in the absence of any commercial or financial relationships that could be construed as a potential conflict of interest.
